# MYB transcription factors in plant cold stress adaptation: integrating phylogeny, signaling networks, and metabolic regulation

**DOI:** 10.1007/s12298-026-01798-0

**Published:** 2026-07-24

**Authors:** Hao Zhang, Yao Yao, Bing Liu, Xiuyun Wang, Yiping Xia, Hong Zhou

**Affiliations:** 1 Genomics and Genetic Engineering Laboratory of Ornamental Plants, Department of Horticulture, College of Agriculture and Biotechnology, Zhejiang University, 866 Yuhangtang Road, Hangzhou, 310058 Zhejiang People’s Republic of China; 2 Department of Plant Pathology, Washington State University, Pullman, WA 99163 USA

**Keywords:** Low temperature, Transcriptional regulatory network, Phylogenetic analysis, Metabolic reprogramming, Post-translational modifications, Epigenetic regulation

## Abstract

MYB transcription factors are pivotal regulators of plant cold acclimation, yet current knowledge about cold-tolerance-related MYBs (crMYBs) remains fragmented across species, with their phylogenetic relationships, regulatory mechanisms, and functional divergence poorly integrated. This review synthesizes functionally validated crMYBs within a phylogenetic framework to provide an updated perspective on their regulatory roles in plant cold acclimation. We curated 106 experimentally validated crMYBs from 45 plant species and systematically characterized their subfamily distribution, regulatory polarity, pathway associations, validation strategies, and cross-species functional patterns. R2R3-MYBs constitute the majority of characterized crMYBs, whereas 1R-MYBs and 3R-MYBs remain underrepresented despite their established links to circadian regulation, cell-cycle control, and stress adaptation. Comparative analyses further indicate that orthologous MYBs retain conserved functions or undergo functional divergence across species. Current evidence implicates crMYBs in multiple regulatory layers of cold acclimation, including ABA-associated responses, CBF/COR-related transcriptional regulation, hormonal crosstalk, osmoprotectant accumulation, phenylpropanoid metabolism, cuticular wax biosynthesis, post-translational modifications, and chromatin-level regulation. Characterization of underexplored 1R-MYB and 3R-MYB members, phylogeny-guided cross-species functional validation, clarification of the mechanisms underlying regulatory polarity divergence, and evaluation of MYB functions under combined stress conditions would contribute substantially to a more comprehensive understanding of plant cold resilience.

**Supplementary Information:**

The online version contains supplementary material available at 10.1007/s12298-026-01798-0.

## Introduction

Climate change and global warming have intensified the frequency and severity of diverse abiotic stresses, including drought, heatwaves, cold spells, and flooding, posing escalating threats to global food security and agricultural sustainability (Zandalinas et al. 2021). Among these challenges, low-temperature stress severely constrains plant growth, development, and geographical distribution. Both chilling injury (0–15 °C) and freezing injury (< 0 °C) inflict substantial losses on crop yield and quality. To cope with these adverse conditions, plants have evolved sophisticated mechanisms to perceive, transduce, and respond to cold signals, thereby enhancing their inherent tolerance (Xin and Browse 2000). Elucidating the molecular underpinnings of these adaptive strategies holds substantial promise for developing climate-resilient crops and ensuring sustainable food production (Ming et al. 2023).

Upon exposure to low temperatures, plants activate intricate regulatory networks responsible for cold signal perception and transduction, wherein transcription factors (TFs) serve as pivotal orchestrators (Peng et al. 2025a, b). Multiple TF families, including AP2/ERF (Ritonga et al. 2021), WRKY (Li et al. 2020), NAC (Xiong et al. 2025) MYB (Wang et al. 2021), and bZIP (Yang et al. 2025), are induced under cold stress to modulate downstream gene expression and enhance freezing tolerance. Within this complex signaling architecture, MYB TFs function as central hubs for integrating endogenous cues with environmental inputs, thereby coordinating the trade-off between growth and stress adaptation (Zhang et al. 2025).

Unlike the well-defined MYB regulatory mechanisms in anthocyanin biosynthesis, MYB functions under cold stress are considerably more diverse. Early studies linked specific MYB members to canonical cold-signalling pathways: *Arabidopsis AtMYB15* interacts with *ICE1* to negatively regulate CBF expression and freezing tolerance (Agarwal et al. 2006). Subsequent work revealed wheat (*Triticum aestivum*) *TaMYB2A* functions in ABA-dependent cold signaling, while another cold-tolerance-related MYB (crMYB) *TaMYB19* bridges ABA-dependent and -independent pathways. Concurrently, MYB-mediated metabolic defense has attracted increasing attention; for instance, *OsMYB4* overexpression promotes the accumulation of osmoprotectants to enhance cold tolerance (Pasquali et al. 2008). More recently, research has expanded to post-translational modifications and chromatin-level regulation. For example, the MaMYB4–MaHDA2 module modulates histone acetylation to affect fatty acid desaturase gene expression and membrane lipid unsaturation (Song et al. 2019), whereas changes in *ScMYB7* promoter methylation regulate its own expression and the CBF pathway under low temperature (Liu et al. 2025).

These findings indicate that MYBs appear to serve as multilayered regulatory nodes. However, most existing studies focus on specific species, individual MYB members, or discrete physiological processes, leaving the evidence fragmented. Inconsistent MYB nomenclature and scattered functional annotations across species further hinder cross-species comparisons and functional inference of orthologs. Additionally, although 1R-MYBs integrate circadian and cold signalling (Sorkin et al. 2023) and 3R-MYBs participate in abiotic stress responses (Dai et al. 2007), research is still dominated by the R2R3-MYB subfamily. Especially for woody perennials, cold-adaptation mechanisms are likely more complex than those in model plants such as *Arabidopsis*, and MYB-mediated regulatory networks may exhibit distinct tissue specificity and seasonal dynamics (Wisniewski et al. 2018). Therefore, a systematic, macroscopic perspective is needed to examine the functional landscape of MYBs under cold stress. To our knowledge, existing MYB reviews have primarily focused on anthocyanin and flavonoid biosynthesis (Liu et al. 2015; Xu et al. 2015; Ma and Constabel 2019), pan-stress responses (Li et al. 2019a, b, c; Pratyusha and Sarada 2022; Bhatt et al. 2025), or developmental regulation (Ambawat et al. 2013; Nguyen and Lee 2016; Peng et al. 2025a, b), whereas a dedicated synthesis specifically addressing MYB-mediated low-temperature responses and cold-adaptation mechanisms remains lacking. Therefore, a family-level synthesis that integrates phylogenetic and cross-species comparative perspectives is needed to systematically clarify the MYB-mediated cold-response network.

To facilitate a more integrated understanding of MYB-mediated cold adaptation, this review synthesizes current knowledge on crMYBs. We curate 106 experimentally validated crMYBs and perform a comparative phylogenetic analysis against the *Arabidopsis* MYB repertoire to consolidate disparate findings and enable cross-species functional inference. We further summarize how MYB transcription factors participate in major cold signaling pathways, metabolic reprogramming, and post-translational and epigenetic regulatory mechanisms during cold acclimation. Together, these analyses offer an updated perspective on the hierarchical regulation of plant freezing tolerance and highlight the potential of MYB-targeted strategies for enhancing crop resilience to low-temperature stress.

## Phylogenetic insights into cold-tolerance-related MYBs

The MYB TF family represents one of the largest transcription factor families in the plant kingdom (Wu et al. 2022). Members of this family are characterized by a modular architecture comprising a highly conserved N-terminal DNA-binding domain and a structurally diverse C-terminal region that mediates regulatory specificity. The N-terminal domain contains one to four imperfect tandem repeats termed the MYB repeat (R1, R2, or R3), each forming a helix-turn-helix motif that facilitates sequence-specific DNA recognition. Based on the number of MYB repeats, plant MYB proteins are classified into four subfamilies: 1R-MYB (MYB-related), R2R3-MYB (2R-MYB), R1R2R3-MYB (3R-MYB), and 4R-MYB (Ogata et al. 1995; Stracke et al. 2001). This repeat-based classification provides a robust phylogenetic framework for comparative functional analyses across species (Lei et al. 2008; Niu et al. 2016).

To systematically identify functionally validated MYB regulators of cold stress responses, we conducted a comprehensive literature search in the Web of Science Core Collection using the Boolean query: (“cold*” OR “chilling*” OR “freezing*”) AND “MYB”. Following duplicate removal, 823 publications were initially retained. Subsequent manual curation based on stringent criteria—specifically, experimental validation through transient or stable transgenic approaches—narrowed the dataset to 101 studies. From these, we compiled 106 crMYBs with demonstrated roles in freezing or chilling tolerance. Full-length protein sequences were retrieved for 101 crMYBs from public databases (NCBI, Phytozome, or Ensembl Plants) or supplementary materials of the original publications; the remaining five sequences were unavailable due to incomplete data deposition. Subfamily classification of all 106 crMYBs was verified through conserved domain analysis using the original study annotations and cross-referenced with InterProScan.

For phylogenetic reconstruction, the 101 crMYBs with available sequences were combined with the complete set of *Arabidopsis thaliana* MYB proteins (AtMYBs) as a reference set. Multiple sequence alignment of full-length protein sequences was performed using MAFFT-L-INS-i (v7.520) with default parameters via the NGPhylogeny.fr web server (Lemoine et al. 2019). A maximum likelihood phylogeny was subsequently inferred using FastTree (v2.1.11) with 20 rate categories and 1000 bootstrap replicates to assess node support. The resulting tree was visualized and annotated with iTOL v6 (Letunic and Bork 2024) and Adobe Illustrator CC 2019 for publication-quality output.

**Fig. 1:**
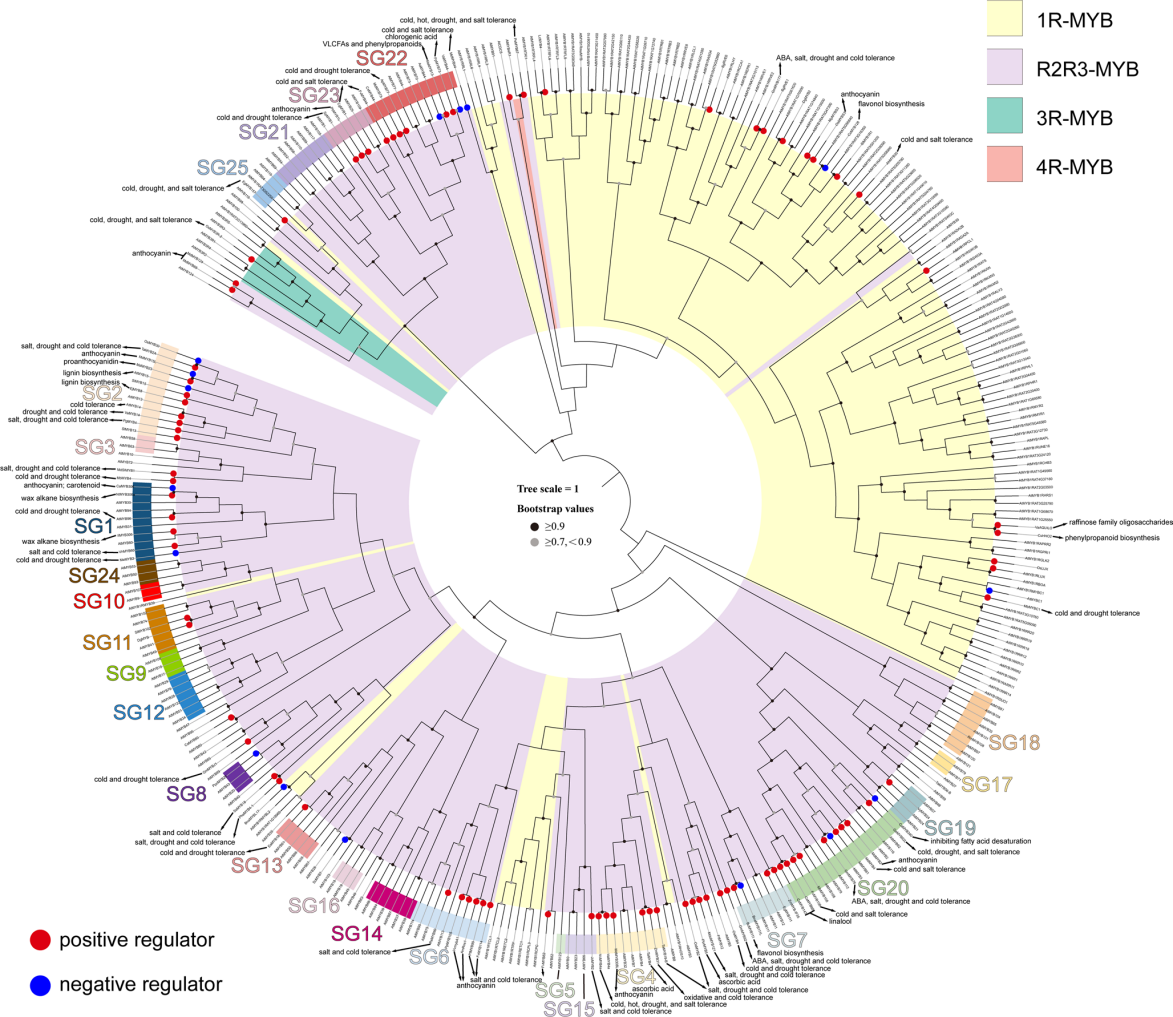
Phylogenetic tree of crMYBs and *Arabidopsis* AtMYBs crMYBs sequences: See Supplementary Table S1. *Arabidopsis* AtMYBs sequences: Sourced from The *Arabidopsis* Information Resource (TAIR) online database. The four MYB subfamilies are indicated by colored blocks in the inner circle of the tree, while the 25 R2R3-MYB subgroups are marked by colored labels adjacent to the corresponding clades. CrMYBs are annotated in the tree: red dots indicate positive regulators of cold tolerance, whereas blue dots indicate negative regulators. Additional experimentally validated biological functions of these crMYBs, apart from cold tolerance regulation, are also indicated beside the corresponding genes. The SVG version of the phylogenetic tree is provided in Supplementary Fig. S1

Phylogenetic analysis revealed that the 101 crMYBs with available sequences are interspersed among *Arabidopsis* MYB proteins rather than forming species-specific clades, indicating substantial sequence conservation and potential functional orthology across diverse plant lineages. Subfamily distribution analysis of all 106 curated crMYBs showed a pronounced bias toward the R2R3-MYB subfamily, comprising 83.0% (88/106) of the total, followed by 1R-MYB (16.0%, 17/106) and 3R-MYB (0.9%, 1/106). No crMYBs were identified within the 4R-MYB subfamily. This uneven distribution reflects both the evolutionary expansion of R2R3-MYBs in vascular plants and a prevailing research focus on this subfamily. These phylogenetic patterns highlight conserved regulatory modules that may represent core components of the cold acclimation network, providing an evolutionary framework for dissecting the mechanistic basis of plant freezing tolerance (Fig. 1).

The curated 106 crMYBs (see Supplementary Table S1; references for curated crMYBs are listed in Supplementary File 2) originate from 45 plant species: 21 from 9 monocots and 85 from 36 dicots. The most represented species are apple (11), tomato (7), and grapevine (7). *Arabidopsis* homologs show uneven citation frequency: *AtMYB44* and *AtMYB4* are each associated with 7 distinct crMYBs, suggesting their prominence as functional paradigms.

Functional characterization reveals a strong positive regulatory bias: 84.9% (90/106) enhance cold tolerance, whereas 15.1% (16/106) suppress it. Pathway involvement is distributed across ABA signaling (47.2%, 50/106), the CBF cascade (71.7%, 76/106), and phenylpropanoid metabolism (22.6%, 24/106), with substantial overlap among these categories.

Validation strategies vary by transformation system: homologous transient expression (25.5%, 27/106), homologous stable transformation (30.2%, 32/106)—primarily in model and herbaceous species, with apple and grapevine as the only woody representatives—and heterologous expression (62.3%, 66/106). Notably, these categories are not mutually exclusive, as some crMYBs were validated through multiple approaches.

### R2R3-MYB

In *Arabidopsis*, the R2R3-MYB subfamily comprises 25 phylogenetically defined subgroups (SGs) based on conserved motif arrangements and functional characteristics (Dubos et al. 2010). Members within the same SG generally share similar domain architectures and regulatory functions, providing a robust framework for functional inference across species. Among the 88 R2R3-crMYBs identified in this study, 81 possessed full-length sequences suitable for phylogenetic placement. Of these, 62 (76.5%) clustered into 14 established SGs, while 19 (23.5%) remained unassigned to any defined subgroup. The most prominently represented subgroups were SG20 (11 members), SG2 (11), SG22 (8), SG1 (6), SG4 (6), SG6 (6), and SG7 (4). A total of 11 SGs (SG3, SG5, SG9, SG10, SG12, SG14, SG16, SG17, SG18, SG21, and SG24) contained no crMYBs, possibly due to incomplete functional annotation and underrepresentation in existing studies.

Comparative analysis reveals that orthologous MYBs involved in low-temperature responses exhibit remarkable conservation in core regulatory functions, particularly in their integration with hormone signaling pathways. This similarity provides a reliable basis for functional inference across diverse plant lineages. A striking example is SG22, which contains seven crMYBs orthologous to *Arabidopsis AtMYB44*. Functional characterization in diverse species—including kiwifruit (*Actinidia arguta*) *AaMYB44*, tea plant (*Camellia sinensis*) *CsMYB44*, apple (*Malus domestica*) *MdMYB44*, and grapevine (*Vitis amurensis*) *VaMYB44*—demonstrates that these orthologs consistently modulate cold tolerance through ABA-dependent signaling. This functional conservation aligns with the *Arabidopsis* paradigm, where *AtMYB44* is constitutively expressed and rapidly induced by multiple abiotic stresses, including cold (Jung et al. 2008). Mechanistically, *AtMYB44* directly interfaces with early ABA signaling by physically interacting with the *PYL8* receptor (Jaradat et al. 2013), suggesting that SG22 MYBs may represent conserved nodes in stress-ABA crosstalk.

Similarly, SG20 encompasses five crMYBs clustering with *Arabidopsis AtMYB2*, including soybean (*Glycine max*) *GmMYB76* (Liao et al. 2008), alfalfa (*Medicago truncatula*) *MtMYB3* and *MtMYB61* (Zhang et al. 2016), and apple *MdMYB2* (Jiang et al. 2022). These orthologs similarly function within ABA-dependent cold response pathways. In *Arabidopsis*, *AtMYB2* acts as a transcriptional activator of ABA-inducible genes, binding to conserved MYB recognition sequences in stress-responsive promoters (Abe et al. 2003). The convergent recruitment of SG20 and SG22 members across phylogenetically distant species underscores the central importance of ABA-mediated transcriptional networks in plant cold acclimation, while subtle differences in regulatory hierarchies likely reflect fine-tuning to species-specific ecological niches.

Conversely, while core functions are often maintained, evolutionary divergence can drive orthologous MYBs toward functionally antagonistic roles, enabling plants to fine-tune conserved signaling pathways such as the CBF cascade to diverse environments. In SG2, three crMYBs are orthologous to *Arabidopsis AtMYB15*, a negative regulator of *CBF* expression (Agarwal et al. 2006). Apple *MdMYB15L* maintains this repressive function (Xu et al. 2018), whereas tomato (*Solanum lycopersicum*) *SlMYB15* has acquired an opposing activator role (Zhang et al. 2020a, b). This reversal likely reflects adaptive selection during plant evolution (Tohge and Fernie 2020). Similar divergence occurs across other subgroups. In SG1, Japanese iris (*Iris laevigata*) *IlMYB306* and cassava (*Manihot esculenta*) *MeMYB2*, both orthologous to *AtMYB60*, act as positive and negative regulators, respectively (Ruan et al. 2017; Yang et al. 2022). Similarly, in SG7, Chinese cabbage (*Brassica campestris* ssp. chinensis) *BcMYB111* and rapeseed (*Brassica napus*) *BnaMYB111L*, both *AtMYB111* orthologs, exhibit opposing effects on cold tolerance (Yao et al. 2020; Chen et al. 2023). These patterns highlight substantial evolutionary plasticity in MYB-mediated cold tolerance, providing adaptive flexibility across heterogeneous thermal environments.

### 1R-MYB

Among the 17 identified 1R-crMYBs, representing 13 plant species, only two (tea plant and grapevine) are woody perennials. Functional characterization reveals a predominant positive regulatory role: 13 act as enhancers of cold tolerance, while four function as negative regulators. The 1R-crMYBs primarily include TFs from classes such as RVE (REVEILLE), HRS1/HHO, LUX (LUX ARRHYTHMO/PHYTOCLOCK), and CCA1 (CIRCADIAN CLOCK ASSOCIATED1).

RVE genes link circadian rhythms (Hughes et al. 2024) to cold acclimation. The 1R-crMYB subset includes three RVE members: fine-stem stylo (*Stylosanthes guianensis*) *SgRVE1* (Wang et al. 2024a, b, c), *SgRVE6* (Chen et al. 2020), and soybean *GmMYB177* (Liao et al. 2008). In *Arabidopsis*, *AtRVE8* binds the Evening Element (EE) motif in the *AtCBF3* promoter to activate downstream cold-responsive genes, while *AtCOR27/AtCOR28* specifically repress *AtRVE8* activity during nighttime, balancing growth with freezing tolerance (Sorkin et al. 2023).

HRS1/HHO-type TFs coordinate nutrient homeostasis and cold responses (Li et al. 2021). Cucumber (*Cucumis sativus*) CsHHO2 enhances fruit cold storage tolerance by regulating glycine-rich RNA-binding protein 3 (*CsGR-RBP3*) (Wang et al. 2024a, b, c), while grapevine *VaAQUILO* (an HRS1-like gene) improves freezing tolerance by promoting osmoprotectant accumulation (Sun et al. 2018).

LUX genes integrate clock function with stress adaptation. *Arabidopsis AtLUX1* maintains circadian rhythms (Huang and Nusinow 2016) and is positively regulated by *AtCBF1* to enhance freezing tolerance (Chow et al. 2014). Rice (*Oryza sativa*) *OsLUX* contains DREB-binding motifs in its promoter, confirming its position downstream of CBFs in the cold response hierarchy (Huang et al. 2023).

*CCA1* is a core component of the *Arabidopsis* circadian clock (Wang and Tobin 1998). In *Arabidopsis*, low temperature suppresses *AtCCA1* alternative splicing, relieving *AtCCA1β*-mediated inhibition of *AtCCA1α* to enhance freezing tolerance (Park et al. 2012). Tea plant *CsMYB128* forms a negative feedback loop with *CsCBF1*: *CsMYB128* represses *CsCBF1* expression, while *CsCBF1* reciprocally activates *CsMYB128*, potentially balancing growth and cold acclimation (Yu et al. 2025a, b).

Despite fewer characterized members compared to R2R3-MYBs, the 1R-MYB subfamily appears integral to cold stress regulation through its unique coupling of circadian/growth rhythms with temperature responses. The functional conservation of this regulatory architecture across diverse species suggests an ancient and indispensable role in plant environmental adaptation.

### 3R-MYB

The 3R-MYB subfamily contains relatively few members, with only one crMYB identified in this study. Plant 3R-MYBs are classified into three groups (A, B, and C) and primarily regulate cell cycle progression during the G2/M phase (Ito 2005). Notably, Group C members respond to abiotic stresses in both monocots and dicots, suggesting a role in coordinating cell cycle dynamics with environmental stress responses (Feng et al. 2017). The sole 3R-crMYB, rice *OsMYB3R-2*, exemplifies this functional integration: it enhances cold tolerance through CBF-dependent signal transduction (Dai et al. 2007).

## Mechanistic insights into myb-mediated cold stress tolerance

### Transcriptional dynamics of MYBs in response to low temperature

Low-temperature stress triggers extensive transcriptional reprogramming across plant species. Comparative transcriptome analyses have identified numerous cold-responsive genes in diverse taxa, including model species (*Arabidopsis*: Jeon and Kim 2013; Klepikova et al. 2019), staple crops (maize: Sobkowiak et al. 2014; Di Fenza et al. 2017; rice: Shen et al. 2014; Da et al. 2017; Xu et al. 2018), fruits (grapevine: Da et al. 2005), and ornamentals (yellowhorn: Wang et al. 2020a, b; birch: Yan et al. 2020). In rice cultivars SQSL and XZX45 exposed to 8°C for 5 days, transcriptome analysis identified 62 differentially expressed MYB/MYB-related genes, outnumbering WRKY (51), NAC (48), and bHLH (47) family members (Yan et al. 2023). In common bean (*Phaseolus vulgaris* L.), comparative transcriptome profiling of the cold-tolerant cultivar ‘Wei Yuan’ and the cold-sensitive cultivar ‘Bai Bu Lao’ under 5°C (0, 6, and 24 h) revealed 98 differentially expressed MYB/MYB-related genes, exceeding the counts for RLK (93), AP2 (76), bHLH (71), WRKY (64), and NAC (56) regulatory genes (Tang et al. 2025).

Expression profiling reveals species-specific variation in MYB regulatory directionality. In *Arabidopsis*, 93 *AtMYBs* were up-regulated and 88 down-regulated under cold stress (Katiyar et al. 2012). In ginger (*Zingiber officinale*), 39 of 231 *ZoMYBs* were induced while 49 were repressed (Xing et al. 2024). In *Dendrobium catenatum*, cold treatment for 24 h up-regulated 28 and down-regulated 18 of 133 *DcMYBs* (Zhang et al. 2021).

Comparative germplasm analyses further link MYB induction to cold-tolerant phenotypes. In some spieces, cold-tolerant genotypes often exhibit more pronounced MYB up-regulation than sensitive counterparts. In cabbage (*Brassica rapa*), comparative expression profiling of the cold-tolerant line ‘Chiifu’ and the cold-sensitive line ‘Kenshin’ revealed contrasting MYB induction patterns: 67 R2R3-MYB and 43 MYB-related genes were preferentially up-regulated in ‘Chiifu’, whereas 49 R2R3-MYB and 46 MYB-related genes exhibited stronger induction in ‘Kenshin’ (Saha et al. 2016). In peanut (*Arachis hypogaea*), 18 MYBs were identified among the transcription factors with significantly higher cold-induced expression in the tolerant cultivar ‘NH5’ than in the sensitive cultivar ‘FH18’ (Jiang et al. 2020). Similarly, in melon (*Cucumis melo*), several MYB TFs were significantly up-regulated under cold stress in the tolerant line ‘162’, whereas their induction was comparatively weaker in the sensitive line ‘13-5A’ (Diao et al. 2024). These patterns collectively suggest a positive association between MYB transcriptional responsiveness and inherent cold tolerance across diverse germplasm, implicating the potential involvement of MYB family members in the divergence of cold adaptation strategies.

### Integration of MYBs into cold signal transduction networks

**Fig. 2:**
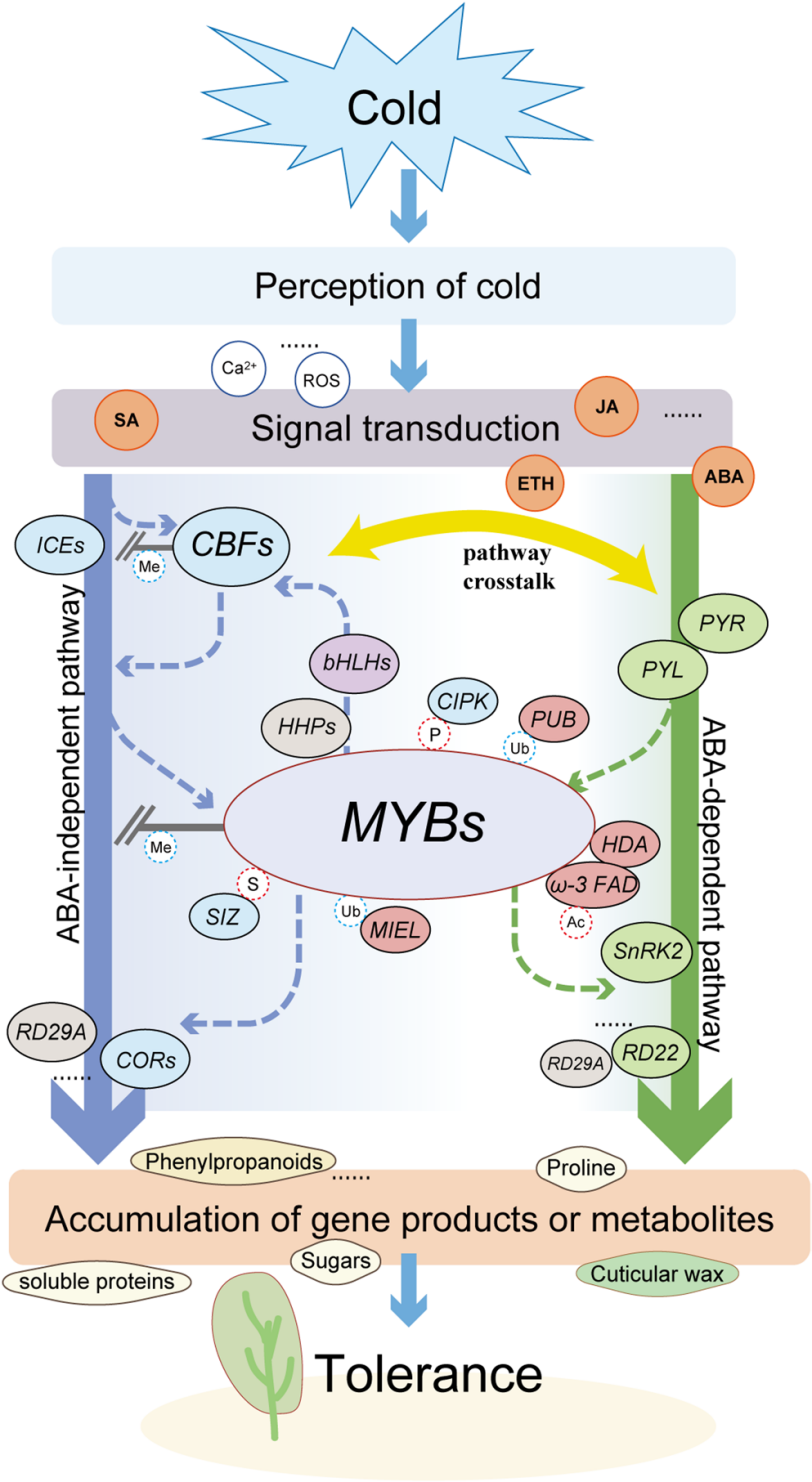
Schematic diagram of MYB TFs as key components in transcriptional regulatory networks during cold stress Low-temperature signals are perceived and transmitted through Ca^2+^ influx and signaling molecules such as ABA, SA, JA, and ETH. MYB transcription factors participate in both ABA-dependent and ABA-independent regulatory pathways. In the ABA-dependent pathway, certain MYBs interact with PYR/PYL receptors and regulate the expression of ABA signaling and responsive genes, including SnRK2 and RD22. In the ABA-independent pathway, MYBs primarily function through the ICE-CBF-COR cascade and its associated regulatory networks. In addition, MYB activity and function are subject to multilayered regulation at both post-translational and epigenetic levels. At the post-translational level, CIPK-mediated phosphorylation (**P**), MIEL/PUB-mediated ubiquitination (**Ub**), and SIZ-mediated SUMOylation (**S**) can directly affect MYB stability and activity. At the epigenetic level, promoter DNA methylation (**Me**) may repress MYB gene expression. Moreover, MYBs themselves can recruit histone deacetylases and methyltransferases to regulate histone acetylation (**Ac**) and histone methylation (**Me**), respectively, thereby remodeling chromatin states at target loci and coordinately regulating cold responses. Ultimately, these regulatory processes are closely associated with stress-adaptive responses, including the accumulation of phenylpropanoid-derived metabolites (e.g., flavonoids and anthocyanins), osmoprotectants (e.g., soluble proteins, soluble sugars, and proline), and cuticular waxes, collectively contributing to plant adaptation to low-temperature environments. This schematic model was constructed based on representative studies discussed throughout Sect.  2.2–2.4 and represents a conceptual synthesis of currently available evidence. Given the diversity of MYB functions among species, specific regulatory relationships may vary across different taxa

Cold tolerance represents a complex quantitative trait governed by interconnected regulatory networks and metabolic pathways (Theocharis et al. 2012; Wu et al. 2016). Based on their reliance on ABA signaling, cold response pathways are broadly classified as ABA-dependent or ABA-independent. The former are typically associated with ABA accumulation and ABRE-mediated transcriptional activation, whereas the latter are primarily mediated through DRE/CRT cis-elements and DREB/CBF-type regulators (Roychoudhury et al. 2013). MYB transcription factors participate extensively in the crosstalk between these pathways, integrating hormonal and environmental cues to orchestrate cold acclimation. Given the complexity of the regulatory processes involved, Fig. 2 presents a hierarchical conceptual framework integrating the above regulatory modules.

#### ABA-dependent regulation

In this pathway, MYBs often serve as direct targets of ABA signaling. The wheat (*Triticum aestivum*) crMYB *TaMYB2A* contains ABRE cis-elements in its promoter and is strongly induced by ABA treatment, suggesting direct regulation by ABA-responsive factors (Mao et al. 2011). Tomato *SlMYB15* directly activates the ABA biosynthesis gene *SlNCED1* and the signaling component *SlABF4*, thereby reinforcing hormone production and signal transduction (Zhang et al. 2022a, b). Oil palm (*Elaeis guineensis*) *EgMYB111* interacts physically with ABA receptors *EgPYR1* and *EgPYL9*, subsequently activating key pathway kinases *EgSnRK2.1*, *EgSnRK2.3*, and *EgSnRK2.5* to regulate downstream cold-responsive genes (Zhou et al. 2022).

#### ABA-independent regulation and CBF crosstalk

Plants predominantly rely on the C-repeat binding factor (*CBF*) cascade for ABA-independent cold responses (Roychoudhury et al. 2013). The central role of CBFs in freezing tolerance is conserved across species (Winfield et al. 2010). crMYBs engage with this cascade through multiple mechanisms: Maize (*Zea mays*) *ZmMYB31* overexpression in *Arabidopsis* up-regulates *AtCBF1*, *AtCBF2*, *AtCBF3*, and *AtGSTU5* under both control and cold conditions (Li et al. 2019a, b, c). Tomato *SlMYB102* activates *SlCBF1*, *SlCBF3*, *SlICE1*, and *SlDREB* genes (Wang et al. 2020a, b), while *SlMYB15* binds directly to type II MYB recognition sites in *SlCBF1-3* promoters (Zhang et al. 2020a, b). Apple *MdMYB23* activates *MdCBF1* and *MdCBF2* (An et al. 2018), and *MdMYB15L* interacts with *MdbHLH33* to enhance *MdCBF2* expression (Xu et al. 2018). *Arabidopsis AtMYB96* activates HEPTAHELICAL PROTEIN (*HHP*), which in turn modulates CBF upstream regulators (Lee and Seo 2015). These examples illustrate direct promoter binding, as well as indirect regulation through interacting partners such as bHLH and HHP proteins, collectively amplifying CBF-dependent cold signals.

#### MYB functions beyond the CBF pathway

Some crMYBs enhance freezing tolerance through CBF-independent routes. Apple *MdMYB88* and *MdMYB124* regulate cold tolerance both via *MdCCA1*-mediated CBF modulation and through CBF-independent activation of COLD SHOCK DOMAIN PROTEIN 3 (*MdCSP3*) (Xie et al. 2018). In rice, *OsMYBS3* confers cold tolerance through high-level constitutive expression yet suppresses the CBF pathway. The temporal distinction—rapid transient CBF response versus slower sustained *OsMYBS3* induction—suggests these factors operate sequentially and complementarily during short-term and prolonged cold stress (Su et al. 2010).

#### Pathway integration and hormone crosstalk

ABA-dependent and ABA-independent pathways are not mutually exclusive; rather, they form an interconnected network enabling robust environmental adaptation (Riera 2005; Barrero et al. 2006; Yoshida et al. 2014). Overexpression of wheat *TaMYB19* in *Arabidopsis* simultaneously activates *AtRD29A* (ABA-independent) and *AtRD22*/*AtMYB2* (ABA-dependent), demonstrating dual-pathway coordination (Zhang et al. 2014). Notably, *RD29A* exemplifies this crosstalk at the promoter level, where DRE/CRT and ABRE elements are interdependent for full stress-responsive activation, with DREB and AREB factors acting synergistically (Narusaka et al. 2003).

#### Beyond ABA, other hormones modulate MYB-mediated cold responses

Salicylic acid (SA) and cold stress synergistically induce tobacco (*Nicotiana tabacum*) *NtMYB306a*, which enhances freezing tolerance when overexpressed (Yu et al. 2022). In peach (*Prunus persica*), methyl jasmonate (MeJA) up-regulates *PpMYB105*, which subsequently activates methionine sulfoxide reductase A1 (*PpMsrA1*) to improve fruit cold storage tolerance (Jiao et al. 2023). Grapevine *VaMYB44*, despite negatively regulating cold tolerance, shows rapid transient induction by ethylene (ETH), ABA, and MeJA (Zhang et al. 2022a, b), highlighting complex hormonal inputs into MYB regulatory nodes.

### Metabolic reprogramming via MYB-regulated defense pathways

#### Primary metabolites and osmoprotection

During cold acclimation, plants accumulate compatible solutes that serve dual functions: protecting cellular membranes from freeze-induced damage and providing respiratory substrates upon recovery. Free sugars, particularly glucose and sucrose, represent prominent osmoprotectants in this context (Bohnert and Jensen 1996; Thomashow 1999). MYBs actively modulate sugar metabolic flux under low temperature. Rice OsMYB4 overexpression elevates glucose, fructose, and sucrose levels in both *Arabidopsis* and apple, correlating with enhanced freezing tolerance (Pasquali et al. 2008). Conversely, tomato *SlMYB102* overexpressants exhibit higher basal soluble sugar content yet show reduced sugar accumulation after cold treatment at 4 °C (Wang et al. 2020a, b). This paradox suggests divergent adaptive strategies: *SlMYB102* may accelerate carbohydrate conversion into protective compounds or respiratory energy, thereby limiting net sugar storage while enhancing metabolic flexibility.

Beyond free sugars, diverse osmoprotectants contribute to cold tolerance. Fine-stem stylo *SgRVE1* overexpression in *Arabidopsis* promotes the accumulation of soluble proteins, sugars, and proline, concomitant with freezing tolerance enhancement (Wang et al. 2024a, b, c). Similarly, grapevine *VaAQUILO* elevates raffinose family oligosaccharide (RFO) content in transgenic *Arabidopsis* and grapevine callus, improving cold tolerance through membrane stabilization (Sun et al. 2018).

#### Phenylpropanoid metabolism and antioxidant defense

Secondary metabolites constitute another critical layer of cold adaptation. The phenylpropanoid pathway generates flavonoids and lignin, which enhance cell wall mechanical strength and scavenge reactive oxygen species (ROS), respectively (Dong and Lin 2021). *Arabidopsis* R2R3-MYB SG7 members—*AtMYB11*, *AtMYB12*, and *AtMYB111*—activate flavonoid biosynthesis in response to low temperature (Mehrtens et al. 2005; Stracke et al. 2007). Their orthologs in *Eucalyptus globulus* (*EgMYB64* and *EgMYB68*) similarly associate with cold tolerance acquisition (Mizrachi et al. 2010). In Chinese cabbage, *BcCBF2*-induced *BcMYB111* binds *BcF3H* and *BcFLS1* promoters, elevating flavonol content and freezing tolerance—an effect validated in both overexpressing hairy roots and heterologously in *Arabidopsis* (Chen et al. 2023).

Within flavonoid metabolism, flavonols and anthocyanins function as antioxidants modulating cellular redox homeostasis and thus protecting the plant from cold-induced ROS damage (Dong and Lin 2021). MYBs serve as master regulators of anthocyanin biosynthesis (Chen et al. 2019). Apple *MdMYB308L* forms a complex with *MdbHLH33*, enhancing the latter’s binding to both *MdCBF2* (cold signaling) and *MdDFR* (anthocyanin synthesis) promoters (An et al. 2020a, b). Radish (*Raphanus sativus*) *RsMYB90*, cold-induced and directly activating *RsCOR78* (CBF pathway) and *RsUFGT* (anthocyanin pathway), exemplifies dual-pathway coordination (Qin et al. 2026). This mechanistic link explains the correlation between anthocyanin accumulation and cold tolerance, though anthocyanin production is not obligatorily required for freezing tolerance, particularly under cold-promoted photoinhibition conditions (Leyva et al. 1995).

Lignin, another phenylpropanoid branch product, reinforces the cell wall and can enhance cold tolerance by improving cellular structural stability and mitigating stress-induced cellular damage under low-temperature conditions (Ramakrishna and Ravishankar 2011). In pear (*Pyrus bretschneideri* var ‘Danxiahong’), six MYB genes are up-regulated during cold storage, which coincides with lignin biosynthetic activation, implicating a NAC-MYB regulatory network in cold-induced cell wall remodeling (Duan et al. 2023). In loquat (*Eriobotrya japonica*), the *EjERF39*–*EjMYB8* complex directly transactivates *Ej4CL1*, thereby enhancing cold-responsive cell wall fortification and fruit cold hardiness (Zhang et al. 2020a, b). Concurrently, *EjMYB1* and *EjMYB2* also participate in low-temperature-induced lignification (Wang et al. 2016).

#### Cuticular wax and physical barrier formation

Beyond phenylpropanoids, cuticular wax alkanes constitute a critical frontline defense. A reinforced wax layer minimizes non-stomatal water loss and shielding cells from cold-induced damage; a reinforced wax layer enhances freezing tolerance (Ni et al. 2014; Lee and Suh 2015). Tobacco *NtMYB306a*, a trichome-specific R2R3-MYB, directly binds promoters of wax alkane synthesis genes (*NtCER1*, *NtCER3*), driving long-chain alkane accumulation and freezing tolerance (Yu et al. 2022). Japanese iris *IlMYB306* similarly enhances cold tolerance through epicuticular wax thickening, coupled with elevated antioxidant activity and proline content (Yang et al. 2022).

### Post-translational modifications and chromatin remodeling of MYBs under cold stress

#### Phosphorylation-mediated activation

Protein phosphorylation can alter transcription factor DNA-binding affinity or interaction specificity within seconds (Kuhn et al. 2024). In plants, cold-induced calcium influx represents one of the earliest signaling events during cold acclimation, activating calcium-dependent and calcium-associated kinases such as CDPKs and CIPKs that subsequently regulate downstream transcription factor activity through phosphorylation (Yuan et al. 2018). Grapevine *VaMYB4a* is phosphorylated by the calcium sensor kinase *VaCIPK18* under low temperature, relieving autoinhibition and activating transcriptional programs that enhance freezing tolerance (Yu et al. 2025a, b). In banana fruit, cold-induced *MaMYB13* interacts with the energy-sensing kinase *MaKIN10* × 1/3 and undergoes phosphorylation. This modification promotes wax and lignin precursor synthesis, reinforcing membrane and cell wall stability against chilling injury (Li et al. 2023).

#### Histone acetylation and chromatin accessibility

MYBs also modulate chromatin states through histone-modifying enzyme recruitment. Banana *MaMYB4* physically interacts with the deacetylase *MaHDA2*, reducing H3 and H4 acetylation at ω-3 fatty acid desaturase (*ω-3 FAD*) promoters. This transcriptional repression modulates membrane lipid unsaturation, optimizing fluidity under low temperature (Song et al. 2019).

Protein stability control through ubiquitination and SUMOylation. E3 ligase-mediated modifications enable transient, switch-like regulation of MYB abundance. Apple *MdMYB308L* is ubiquitinated by the RING-type E3 ligase *MdMIEL1* and degraded via the 26 S proteasome, attenuating anthocyanin accumulation and cold tolerance (An et al. 2020a, b). Conversely, chrysanthemum (*Chrysanthemum × morifolium*) *CmMYB73* undergoes U-box-type E3 ligase (*CmPUB15*)-mediated ubiquitination; its degradation relieves repression of anthocyanin biosynthesis genes, promoting pigment accumulation under cold stress (Geng et al. 2025). In an alternative regulatory mode, apple *MdMYB2* activates the SUMO E3 ligase *MdSIZ1*, which SUMOylates *MdMYB1*. This modification enhances *MdMYB1* stability, facilitating anthocyanin accumulation and freezing tolerance (Jiang et al. 2022).

#### DNA methylation dynamics

Low temperature rapidly alters MYB promoter methylation status, suppressing or enhancing expression. In sugarcane (*Saccharum* spp.), two CpG islands in the *ScMYB7* promoter become hypermethylated after 24 h at 4 °C, repressing *ScMYB7* transcription. This derepresses the CBF pathway, ultimately improving cold tolerance (Liu et al. 2025). Conversely, in tea plant, combined low-temperature and MeJA treatment induces promoter demethylation of *CsMYB68* and *CsMYB147*, elevating their expression. This promotes linalool accumulation and ROS scavenging, enhancing freezing tolerance (Yue et al. 2025).

#### Histone methylation and transcriptional activation

MYBs recruit histone methyltransferases to remodel chromatin at cold-responsive loci. *Arabidopsis AtCDC5* (crMYB) interacts with *AtICE1*, recruiting H3K4me3 methyltransferases to the *AtCBF3* locus. This increases activating H3K4me3 marks and reduces repressive H3K27me3 marks, promoting *AtCBF3* expression and freezing tolerance (Xin et al. 2025). Similarly, chrysanthemum *DgMYB* binds the *DgPOD* promoter and recruits H3K4me3 methyltransferases, elevating H3K4me3 levels to up-regulate peroxidase expression, accelerate ROS scavenging, and improve cold tolerance (Luo et al. 2024).

## Knowledge gaps, regulatory complexity, and future perspectives

Despite extensive progress, the current understanding of MYB-mediated cold tolerance remains incomplete. Functional investigations have concentrated disproportionately on the R2R3-MYB subfamily, while the 1R-MYB and 3R-MYB subfamilies are underexplored. Emerging evidence indicates that 1R-MYBs integrate circadian rhythms with low-temperature signaling (Sorkin et al. 2023), and Group C 3R-MYBs respond to abiotic stresses (Feng et al. 2017), suggesting that a substantial number of uncharacterized crMYBs await discovery. Furthermore, fragmented data deposition across disparate platforms and inconsistent MYB nomenclature—compounded by the lack of unified annotation systems—severely impede cross-species functional inference, despite extensive homologous evolutionary analyses.

The positive regulatory roles of MYBs established in model species should not be uncritically extrapolated to other plants; species-specific experimental validation remains essential to determine regulatory directionality. The observed divergence in functional polarity (activation versus repression) among orthologs indicates that MYB regulatory modes are evolutionarily plastic rather than fixed. Rather than being dismissed as mere exceptions to functional conservation, such polarity reversals should be recognized as key unresolved phenomena that may illuminate how conserved transcription factor families are rewired during species-specific cold adaptation. Future studies should elucidate whether these reversals stem from alterations in cis-regulatory landscapes, interacting protein partners, post-translational modification patterns, DNA-binding specificity, or the hierarchical position of MYBs within cold-responsive regulatory networks. This plasticity may also provide theoretical opportunities for engineering MYB properties through gene editing, enabling more targeted improvement of crop cold tolerance.

**Fig. 3:**
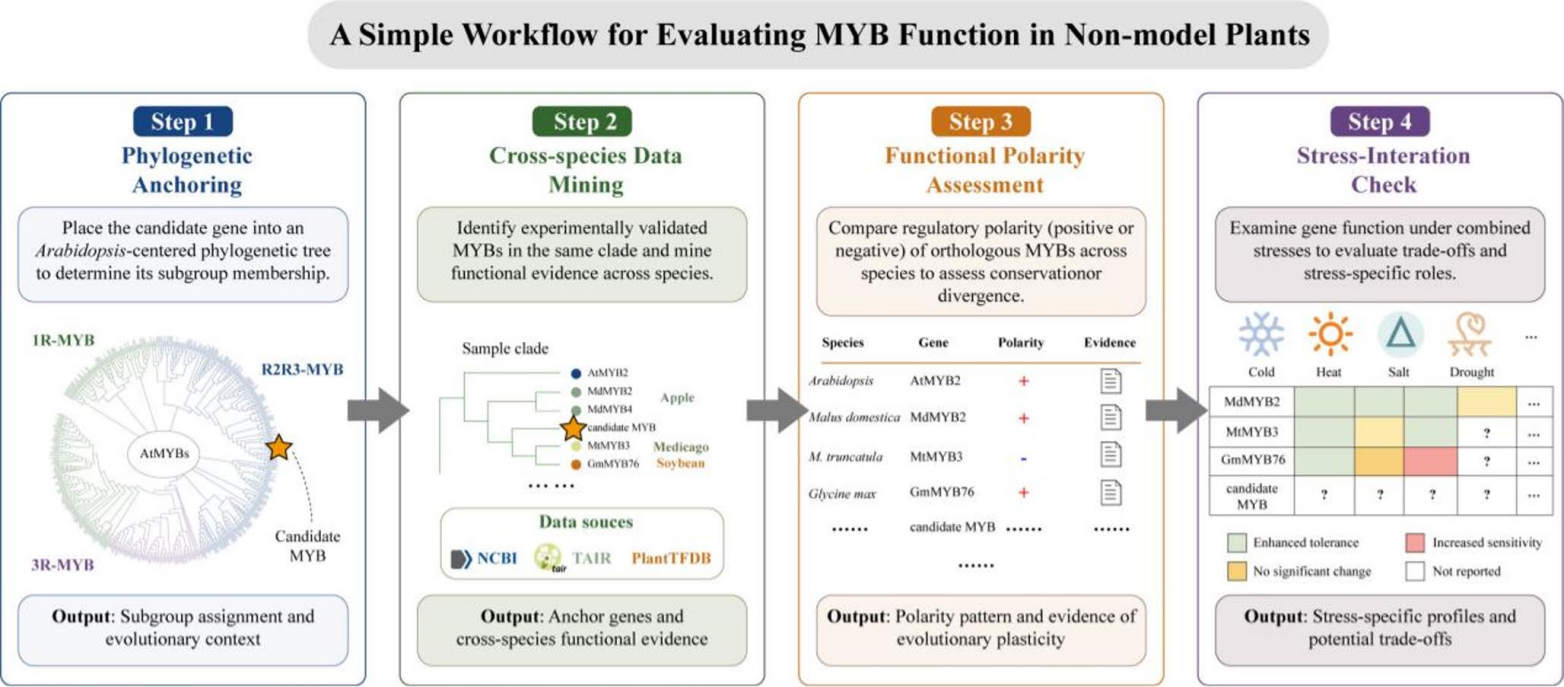
Conceptual workflow for phylogeny-guided functional evaluation of crMYBs in non-model plants. The workflow integrates phylogenetic anchoring, cross-species functional mining, functional polarity assessment, and stress-interaction evaluation to facilitate cross-species functional inference of candidate crMYBs. Gene names and relationships shown in the workflow are provided for illustrative purposes only and do not represent actual functional predictions or validated regulatory relationships

Equally important, MYB functions under combined stresses cannot be predicted from single-stress studies. MYBs exhibit pleiotropic roles across abiotic stress interactions, introducing potential “side effects” with traditional single-gene manipulation strategies (Joseph et al. 2025). In *Dendrobium catenatum*, *DcMYB97* is induced by cold but repressed by heat and salt, whereas *DcMYB80* shows the opposite pattern (Zhang et al. 2021). Similar stress-specific expression divergence occurs in alfalfa R2R3-MYBs (Li et al. 2019a, b, c). Moreover, bamboo (*Phyllostachys edulis*) *PheMYB4-1* enhances cold tolerance yet increases sensitivity to salt and drought (Hou et al. 2018). These findings underscore the necessity of evaluating tolerance mechanisms within the broader context of stress interaction networks (Vinocur and Altman 2005; Pandey et al. 2015).

Thus, constructing a comprehensive evolutionary framework for crMYBs (and, by extension, the entire MYB superfamily) requires broadening the taxonomic scope to support robust comparative analyses across a broader phylogenetic spectrum. Future efforts should prioritize high-resolution comparisons among closely related species, particularly intraspecific cultivars with contrasting cold-adaptation phenotypes. These fine-scale approaches will be essential for elucidating the evolutionary mechanisms underlying MYB functional diversification and for enhancing predictive functional annotation in plant stress biology and crop improvement.

Based on the current synthesis, a phylogeny-guided evaluation workflow may facilitate functional characterization of MYBs in non-model plants and improve cross-species functional inference (Fig. 3). First, candidate MYBs can be phylogenetically anchored within the Arabidopsis-centered MYB framework to determine subgroup affiliation. Second, experimentally validated orthologs within the same clade can be used as reference nodes to guide cross-species functional prediction, thereby reducing information loss caused by inconsistent nomenclature and fragmented annotations. Third, comparative assessment of regulatory polarity among orthologous MYBs may help identify lineage-specific functional divergence and evolutionary plasticity. Finally, evaluation under combined stress conditions should be incorporated to assess potential trade-offs that may not be evident from single-stress experiments alone.

Technological advances in single-cell epigenomics, live-cell imaging, optogenetics, and chemogenetics now provide unprecedented resolution to dissect cold signal transduction from perception through epigenetic reprogramming to transcriptional output. Future research should integrate multi-omics datasets (transcriptomics, proteomics, metabolomics, epigenomics) with machine learning to construct cross-species, developmental stage-specific regulatory networks and quantify individual factor contributions under combined stresses. Precision genome editing tools—including CRISPR/Cas systems (Doudna and Charpentier 2014; Braun et al. 2017; Nuñez et al. 2021), OMEGAoff (Kannan et al. 2025), and LANS-Set2 (Lerner et al. 2020)—will enable reversible modulation of MYB expression, protein modifications, and chromatin states. These approaches hold promise for directed breeding of climate-resilient crops with enhanced low-temperature tolerance, offering sustainable solutions for agricultural production under variable climates (Reul and Chandramohan 2007; Joseph et al. 2025).

## Supplementary Information

Below is the link to the electronic supplementary material.

Supplementary Material 1

Supplementary Material 2

Supplementary Material 3

## Data Availability

All data supporting this review are available from the corresponding author upon reasonable request.
