# Supplementary material for: MYB transcription factors in plant cold stress adaptation: integrating phylogeny, signaling networks, and metabolic regulation
Source: Physiol Mol Biol Plants. 2026 Jul 24;32(8):1687–701. doi: 10.1007/s12298-026-01798-0 (PMC13437875; doi:10.1007/s12298-026-01798-0)
Supplement: Supplementary file 1 [file 12298_2026_1798_MOESM2_ESM.docx]

| **CrMYB** | **References** | **References** |
| --- | --- | --- |
| *BcMYB111* | BcMYB111 Responds to BcCBF2 and Induces Flavonol Biosynthesis to Enhance Tolerance under Cold Stress in Non-Heading Chinese Cabbage | (Chen et al. 2023) |
| *PeMYB87* | Identification of the passion fruit (Passiflora edulis Sims) MYB family in fruit development and abiotic stress, and functional analysis of PeMYB87 in abiotic stresses | (Zhang et al. 2023) |
| *SgRVE1* | Over-expression of SgRVE1 from fine-stem stylo (Stylosanthes guianensis var. intermedia) up-regulates CBF gene expression and enhances cold tolerance in Arabidopsis | (Wang et al. 2024) |
| *SgRVE6* | SgRVE6, a LHY-CCA1-Like Transcription Factor From Fine-Stem Stylo, Upregulates NB-LRR Gene Expression and Enhances Cold Tolerance in Tobacco | (Chen et al. 2020) |
| *JcMYB2* | Genome-wide identification of the Jatropha curcas MYB family and functional analysis of the abiotic stress responsive gene JcMYB2 | (Peng et al. 2016) |
| *FvMYB108* | FvMYB108, a MYB Gene from Fragaria vesca, Positively Regulates Cold and Salt Tolerance of Arabidopsis | (Song et al. 2024) |
| *FvMYB114* | Overexpression of a Fragaria vesca 1R-MYB Transcription Factor Gene (FvMYB114) Increases Salt and Cold Tolerance in Arabidopsis thaliana | (Li et al. 2023) |
| *FvMYB44* | FvMYB44, a Strawberry R2R3-MYB Transcription Factor, Improved Salt and Cold Stress Tolerance in Transgenic Arabidopsis | (Li et al. 2023) |
| *FvMYB82* | Overexpression of a Fragaria vesca MYB Transcription Factor Gene (FvMYB82) Increases Salt and Cold Tolerance in Arabidopsis thaliana | (Li et al. 2022) |
| *CsMYB128* | A CCA1-like MYB subfamily member CsMYB128 participates in chilling sensitivity and cold tolerance in tea plants ( Camellia sinensis) | (Yu et al. 2025) |
| *CsMYB147* | Divergent MYB paralogs determine spatial distribution of linalool mediated by JA and DNA demethylation participating in aroma formation and cold tolerance of tea plants | (Yue et al. 2025) |
| *CsMYB44* | A novel orphan gene CsOG3 drives CsMYB44-dependent cold tolerance in tea plant | (Gao et al. 2025) |
| *CsMYB68* | Divergent MYB paralogs determine spatial distribution of linalool mediated by JA and DNA demethylation participating in aroma formation and cold tolerance of tea plants | (Yue et al. 2025) |
| *GmMYB177* | Soybean GmMYB76, GmMYB92, and GmMYB177 genes confer stress tolerance in transgenic Arabidopsis plants | (Liao et al. 2008) |
| *GmMYB76* | Soybean GmMYB76, GmMYB92, and GmMYB177 genes confer stress tolerance in transgenic Arabidopsis plants | (Liao et al. 2008) |
| *GmMYB92* | Soybean GmMYB76, GmMYB92, and GmMYB177 genes confer stress tolerance in transgenic Arabidopsis plants | (Liao et al. 2008) |
| *GmMYBJ1* | A novel MYB transcription factor, GmMYBJ1, from soybean confers drought and cold tolerance in Arabidopsis thaliana | (Su et al. 2014) |
| *PbrMYB5* | A novel MYB transcription factor regulates ascorbic acid synthesis and affects cold tolerance | (Xing et al. 2019) |
| *RmMYB44* | RmMYB44 Confers Resistance to Chilling, Drought, and Salt Stress in Both Rosa multiflora and Tobacco | (Zhang et al. 2024) |
| *LeAN2* | Overexpression of R2R3-MYB gene leads to accumulation of anthocyanin and enhanced resistance to chilling and oxidative stress | (Meng et al. 2014) |
| *MdSlMYB1* | Overexpression of a R2R3 MYB gene MdSIMYB1 increases tolerance to multiple stresses in transgenic tobacco and apples | (Wang et al. 2014) |
| *SlMYB102* | SlMYB102 expression enhances low temperature stress resistance in tomato plants | (Wang et al. 2020) |
| *SlMYB13* | Jasmonic Acid Activates Transcription Factor SlMYB13 to Enhance Cold Resistance in Tomato | (Zhang et al. 2025) |
| *SlMYB15* | The HY5 and MYB15 transcription factors positively regulate cold tolerance in tomato via the CBF pathway | (Zhang et al. 2020) |
| *SlMYB1R1* | Overexpression of SlMYB1R1 improves chilling stress tolerance in tomato | (Nie et al. 2024) |
| *SpMYB1* | Overexpression of R2R3-MYB type transcription factor SpMYB1 enhances cold and drought tolerance in tomato | (Jin et al. 2025) |
| *EaMYB18* | Isolation and functional validation of stress tolerant EaMYB18 gene and its comparative physio-biochemical analysis with transgenic tobacco plants overexpressing SoMYB18 and SsMYB18 | (Shingote et al. 2020) |
| *ScMYB7* | Cold-Induced Promoter Methylation Attenuates ScMYB7-Mediated Repression of the CBF Pathway: A Proposed Mechanism for Sugarcane Cold Adaptation | (Liu et al. 2025) |
| *SsMYB18* | Ectopic Expression of SsMYB18, a Novel MYB Transcription Factor from Saccharum spontaneum Augments Salt and Cold Tolerance in Tobacco | (Shingote et al. 2017) |
| *CtMYB63* | The CtMYB63-CtU-box1-CtUCH1 module regulates cold tolerance and Hydroxysafflor yellow A accumulation in Carthamus tinctorius | (Hong et al. 2023) |
| *LlMYB3* | A MYB-Related Transcription Factor from Lilium lancifolium L. (LlMYB3) Is Involved in Anthocyanin Biosynthesis Pathway and Enhances Multiple Abiotic Stress Tolerance in Arabidopsis thaliana | (Yong et al. 2019) |
| *AhMYB30* | A MYB-related transcription factor from peanut, AhMYB30, improves freezing and salt stress tolerance in transgenic Arabidopsis through both DREB/CBF and ABA-signaling pathways | (Chen et al. 2023) |
| *CsHHO2* | A cold-inducible MYB like transcription factor, CsHHO2, positively regulates chilling tolerance of cucumber fruit by enhancing CsGR-RBP3 expression | (Wang et al. 2024) |
| *CgMYB1* | CgMYB1, an R2R3-MYB transcription factor, can alleviate abiotic stress in an annual halophyte Chenopodium glaucum | (Zhou et al. 2023) |
| *ApMYB77* | An Acer palmatum R2R3-MYB Gene, ApMYB77, Confers Freezing and Drought Tolerance in Arabidopsis thaliana | (Zhu et al. 2023) |
| *AmRos1* | Overexpression of Rosea1 From Snapdragon Enhances Anthocyanin Accumulation and Abiotic Stress Tolerance in Transgenic Tobacco | (Naing et al. 2018) |
| *DgMYB* | Transcription factor DgMYB recruits H3K4me3 methylase to DgPEROXIDASE to enhance chrysanthemum cold tolerance | (Luo et al. 2024) |
| *DgMYB2* | DgMYB2 improves cold resistance in chrysanthemum by directly targeting DgGPX1 | (Yang et al. 2022) |
| *CaMYB306* | The pepper MYB transcription factor CaMYB306 accelerates fruit coloration and negatively regulates cold resistance | (Ma et al. 2022) |
| *CaMYB340* | The CaMYB340 transcription factor induces chilling injury in post-harvest bell pepper by inhibiting fatty acid desaturation | (Ge et al. 2022) |
| *CaMYB80* | CaMYB80 enhances the cold tolerance of pepper by directly targeting CaPOA1 | (Xiao et al. 2024) |
| *VcMYB4a* | VcMYB4a, an R2R3-MYB transcription factor from Vaccinium corymbosum, negatively regulates salt, drought, and temperature stress | (Zhang et al. 2020) |
| *PbMYB1L* | A novel R2R3-MYB transcription factor PbMYB1L of Pyrus bretschneideri regulates cold tolerance and anthocyanin accumulation | (Zhou et al. 2024) |
| *PpcMYB20* | MYB20, an R2R3-type MYB transcription factor, negatively regulates salt and cold stress tolerance in pears | (Liu et al. 2025) |
| *RsMYB90* | RsMYB90, a R2R3-MYB transcription factor, plays a positive role in regulating low temperature stress in radish | (Qin et al. 2026) |
| *PheMYB4-1* | The R2R3MYB Gene Family in Phyllostachys edulis: Genome-Wide Analysis and Identification of Stress or Development-Related R2R3MYBs | (Hou et al. 2018) |
| *AaMYB44* | Overexpression of the Kiwifruit Transcription Factor AaMYB44 Decreases the Cold Tolerance in Arabidopsis thaliana | (Li et al. 2024) |
| *GaMYB62L* | Functional Characterization of Cotton GaMYB62L, a Novel R2R3 TF in Transgenic Arabidopsis | (Butt et al. 2017) |
| *MeMYB2* | Genome-wide characterization and expression analysis enables identification of abiotic stress-responsive MYB transcription factors in cassava (Manihot esculenta) | (Ruan et al. 2017) |
| *MtMYB3* | Opposing Control by Transcription Factors MYB61 and MYB3 Increases Freezing Tolerance by Relieving C-Repeat Binding Factor Suppression | (Zhang et al. 2016) |
| *MtMYB61* | Opposing Control by Transcription Factors MYB61 and MYB3 Increases Freezing Tolerance by Relieving C-Repeat Binding Factor Suppression | (Zhang et al. 2016) |
| *AtCDC5* | MYB Transcription Factor CDC5 Activates CBF3 Expression to Positively Regulate Freezing Tolerance via Cooperating With ICE1 and Histone Modification in Arabidopsis | (Xin et al. 2025) |
| *AtMYB14* | AtMYB14 Regulates Cold Tolerance in Arabidopsis | (Chen et al. 2013) |
| *AtMYB15* | Phosphorylation of the transcriptional repressor MYB15 by mitogen-activated protein kinase 6 is required for freezing tolerance in Arabidopsis | (Kim et al. 2017) |
| *AtMYB96* | The MYB96–HHP module integrates cold and abscisic acid signaling to activate the CBF–COR pathway in Arabidopsis | (Lee and Seo 2015) |
| *AtMYBC1* | A single-repeat R3-MYB transcription factor MYBC1 negatively regulates freezing tolerance in Arabidopsis | (Zhai et al. 2010) |
| *AtLUX* | Transcriptional Regulation of LUX by CBF1 Mediates Cold Input to the Circadian Clock in Arabidopsis | (Chow et al. 2014) |
| *EjMYB8* | ETHYLENE RESPONSE FACTOR39-MYB8 complex regulates low-temperature-induced lignification of loquat fruit | (Zhang et al. 2020) |
| *MdMYB308L* | An apple MYB transcription factor regulates cold tolerance and anthocyanin accumulation and undergoes MIEL1-mediated degradation | (An et al. 2020) |
| *MdMYB108L* | A feedback loop involving MdMYB108L and MdHY5 controls apple cold tolerance | (Wang et al. 2019) |
| *MdMYB124* | An atypical R2R3 MYB transcription factor increases cold hardiness by CBF-dependent and CBF-independent pathways in apple | (Xie et al. 2018) |
| *MdMYB15L* | Overexpression of a repressor MdMYB15L negatively regulates anthocyanin and cold tolerance in red-fleshed callus | (Xu et al. 2018) |
| *MdMYB2* | The transcription factor MdMYB2 influences cold tolerance and anthocyanin accumulation by activating SUMO E3 ligase MdSIZ1 in apple | (Jiang et al. 2022) |
| *MdMYB23* | R2R3-MYB transcription factor MdMYB23 is involved in the cold tolerance and proanthocyanidin accumulation in apple | (An et al. 2018) |
| *MdMYB4* | MdMYB4, an R2R3-Type MYB Transcription Factor, Plays a Crucial Role in Cold and Salt Stress in Apple Calli | (Wu et al. 2017) |
| *MdMYB44* | Functional characterisation of MdMYB44 as a negative regulator in the response to cold and salt stress in apple calli | (Wu et al. 2018) |
| *MdMYB73* | Functional characterization of MdMYB73 reveals its involvement in cold stress response in apple calli and Arabidopsis | (Zhang et al. 2017) |
| *MdMYB88* | An atypical R2R3 MYB transcription factor increases cold hardiness by CBF-dependent and CBF-independent pathways in apple | (Xie et al. 2018) |
| *MdoMYB121* | Genome wide analysis of the apple MYB transcription factor family allows the identification of MdoMYB121 gene confering abiotic stress tolerance in plants | (Cao et al. 2013) |
| *VaAQUILO* | The GARP/MYB-related grape transcription factor AQUILO improves cold tolerance and promotes the accumulation of raffinose family oligosaccharides | (Sun et al. 2018) |
| *VaMYB14* | Vitis Myb14 confer cold and drought tolerance by activating lipid transfer protein genes expression and reactive oxygen species scavenge | (Fang et al. 2024) |
| *VaMYB44* | VaMYB44 transcription factor from Chinese wild Vitis amurensis negatively regulates cold tolerance in transgenic Arabidopsis thaliana and V. vinifera | (Zhang et al. 2022) |
| *VaMYB4a* | Phosphorylation-dependent VaMYB4a regulates cold stress in grapevine by inhibiting VaPIF3 and activating VaCBF4 | (Yu et al. 2025) |
| *VhMYB60* | Arabidopsis thaliana Plants' Overexpression of the MYB Transcription Factor VhMYB60 in the Face of Stress Hazards Enhances Salt and Cold Tolerance | (Chen et al. 2025) |
| *VvmybA1* | Physiological and molecular responses of 'Hamlin' sweet orange trees expressing the VvmybA1 gene under cold stress conditions | (Mahmoud et al. 2024) |
| *VyMYB24* | Synergistic Interaction Between VyMYB24 from Chinese Wild Grape Vitis yeshanensis and Cytokinin Enhances Cold Tolerance in Transgenic Tobacco | (Li et al. 2025) |
| *RmMYB108* | An R2R3-MYB Transcription Factor RmMYB108 Responds to Chilling Stress of Rosa multiflora and Conferred Cold Tolerance of Arabidopsis | (Dong et al. 2021) |
| *FtMYB12* | Ectopic Expression of Fagopyrum tataricum FtMYB12 Improves Cold Tolerance in Arabidopsis thaliana | (Zhou et al. 2015) |
| *PgMYB4* | Overexpression of the Panax ginseng MYB4 gene enhances stress tolerance in transgenic Arabidopsis thaliana | (Lian et al. 2021) |
| *MbMYB108* | Molecular Cloning and Characterization of MbMYB108, a Malus baccata MYB Transcription Factor Gene, with Functions in Tolerance to Cold and Drought Stress in Transgenic Arabidopsis thaliana | (Yao et al. 2022) |
| *MbMYB4* | Overexpression of a Malus baccata MYB Transcription Factor Gene MbMYB4 Increases Cold and Drought Tolerance in Arabidopsis thaliana | (Yao et al. 2022) |
| *MbMYBC1* | MbMYBC1, a M. baccata MYB transcription factor, contribute to cold and drought stress tolerance in transgenic Arabidopsis | (Liu et al. 2023) |
| *OsLUX* | OsLUX Confers Rice Cold Tolerance as a Positive Regulatory Factor | (Huang et al. 2023) |
| *OsMYB2* | A R2R3-type MYB gene, OsMYB2, is involved in salt, cold, and dehydration tolerance in rice | (Yang et al. 2012) |
| *OsMYB30* | The OsMYB30 Transcription Factor Suppresses Cold Tolerance by Interacting with a JAZ Protein and Suppressing b-Amylase Expression | (Lv et al. 2017) |
| *OsMYB3R-2* | Overexpression of an R1R2R3 MYB Gene, OsMYB3R-2,Increases Tolerance to Freezing, Drought, and Salt Stress in Transgenic Arabidopsis | (Dai et al. 2007) |
| *OsMYB4* | Osmyb4 expression improves adaptive responses to drought and cold stress in transgenic apples | (Pasquali et al. 2008) |
| *OsMYBS3* | WD40 protein OsTTG1 promotes anthocyanin accumulation and CBF transcription factor-dependent pathways for rice cold tolerance | (Zhu et al. 2024) |
| *PpMYB73* | Transcription factor PpMYB73 inhibits PpPPO1 transcription and flesh browning in hydrogen sulfide-mitigated chilling injury of peach fruit | (Zhao et al. 2026) |
| *PpMYB105* | PpMYB105 inhibits chilling injury by regulating PpMsrA1 in peach fruit | (Jiao et al. 2023) |
| *PfMYB90* | Heterologous Expression of a Potential ' Paulownia fortunei' MYB Factor Gene, PfMYB90, Improves Salt and Cold Tolerance in Arabidopsis | (Wang et al. 2024) |
| *MaMYB13* | MaMYB13 is involved in response to chilling stress via activating expression of VLCFAs and phenylpropanoids biosynthesis-related genes in postharvest banana fruit | (Li et al. 2023) |
| *MpMYBS3* | MpMYBS3 as a crucial transcription factor of cold signaling confers the cold tolerance of banana | (Dou et al. 2016) |
| *TaMYB19-B* | Characterization of a Wheat R2R3-MYB Transcription Factor Gene, TaMYB19, Involved in Enhanced Abiotic Stresses in Arabidopsis | (Zhang et al. 2014) |
| *TaMYB2A* | Transgenic expression of TaMYB2A confers enhanced tolerance to multiple abiotic stresses in Arabidopsis | (Mao et al. 2011) |
| *TaMYB4* | Overexpression of TaMYB4 Confers Freezing Tolerance in Arabidopsis thaliana | (Tian et al. 2023) |
| *TaMYB56-B* | Overexpression of a wheat MYB transcription factor gene, TaMYB56-B, enhances tolerances to freezing and salt stresses in transgenic Arabidopsis | (Zhang et al. 2012) |
| *NtMYB306a* | Cloning, characterization and functional analysis of NtMYB306a gene reveals its role in wax alkane biosynthesis of tobacco trichomes and stress tolerance | (Yu et al. 2022) |
| *IlMYB306* | Comparative analysis of R2R3-MYB transcription factors in the flower of Iris laevigata identifies a novel gene regulating tobacco cold tolerance | (Yang et al. 2022) |
| *LcMYB4* | LcMYB4, an unknown function transcription factor gene from sheepgrass, as a positive regulator of chilling and freezing tolerance in transgenic Arabidopsis | (Li et al. 2020) |
| *BnaMYB111L* | The R2R3-MYB transcription factor BnaMYB111L from rapeseed modulates reactive oxygen species accumulation and hypersensitive-like cell death | (Yao et al. 2020) |
| *BnaMYBL17* | Cloning and Functional Characterization of Cold-Inducible MYB-like 17 Transcription Factor in Rapeseed (Brassica napus L.) | (Luo et al. 2023) |
| *EgMYB111* | Interaction of R2R3-MYB transcription factor EgMYB111 with ABA receptors enhances cold tolerance in oil palm | (Zhou et al. 2025) |
| *EgMYB157* | The oil palm R2R3-MYB subfamily genes EgMYB111 and EgMYB157 improve multiple abiotic stress tolerance in transgenic Arabidopsis plants | (Zhou et al. 2022) |
| *ZmMYB31* | ZmMYB31, a R2R3‑MYB transcription factor in maize, positively regulates the expression of CBF genes and enhances resistance to chilling and oxidative stress | (Li et al. 2019) |
| *ZmMYB-IF35* | Overexpression of maize MYB-IF35 increases chilling tolerance in Arabidopsis | (Meng and Sui 2019) |

**References**

An J, Li R, Qu F, You C, Wang X, Hao Y (2018) R2r3-myb transcription factor mdmyb23 is involved in the cold tolerance and proanthocyanidin accumulation in apple. The Plant journal : for cell and molecular biology 96(3):562-577. https://doi.org/10.1111/tpj.14050

An J, Wang X, Zhang X et al (2020) An apple myb transcription factor regulates cold tolerance and anthocyanin accumulation and undergoes miel1-mediated degradation. Plant Biotechnol J 18(2):337-353. https://doi.org/10.1111/pbi.13201

Butt HI, Yang Z, Chen E et al (2017) Functional characterization of cotton gamyb62l, a novel r2r3 tf in transgenic arabidopsis. PLoS One 12(1):e170578. https://doi.org/10.1371/journal.pone.0170578

Cao Z, Zhang S, Wang R, Zhang R, Hao Y (2013) Genome wide analysis of the apple myb transcription factor family allows the identification of mdomyb121 gene confering abiotic stress tolerance in plants. PLoS One 8(7):e69955. https://doi.org/10.1371/journal.pone.0069955

Chen N, Pan L, Yang Z et al (2023) A myb-related transcription factor from peanut, ahmyb30, improves freezing and salt stress tolerance in transgenic arabidopsis through both dreb/cbf and aba-signaling pathways. Front Plant Sci 14:1136626. https://doi.org/10.3389/fpls.2023.1136626

Chen S, Huang H, Chen J et al (2020) Sgrve6, a lhy-cca1-like transcription factor from fine-stem stylo, upregulates nb-lrr gene expression and enhances cold tolerance in tobacco. Front Plant Sci 11:1276. https://doi.org/10.3389/fpls.2020.01276

Chen X, Wu Y, Yu Z et al (2023) Bcmyb111 responds to bccbf2 and induces flavonol biosynthesis to enhance tolerance under cold stress in non-heading chinese cabbage. International journal of molecular sciences 24(10). https://doi.org/10.3390/ijms24108670

Chen Y, Chen Z, Kang J, Kang D, Gu H, Qin G (2013) Atmyb14 regulates cold tolerance in arabidopsis. Plant Mol Biol Report 31(1):87-97. https://doi.org/https://doi.org/10.1007/s11105-012-0481-z

Chen Z, Wang J, Li W et al (2025) Arabidopsis thaliana plants' overexpression of the myb transcription factor vhmyb60 in the face of stress hazards enhances salt and cold tolerance. International journal of molecular sciences 26(4). https://doi.org/10.3390/ijms26041695

Chow BY, Sanchez SE, Breton G, Pruneda-Paz JL, Krogan NT, Kay SA (2014) Transcriptional regulation of lux by cbf1 mediates cold input to the circadian clock in arabidopsis. Current biology : CB 24(13):1518-1524. https://doi.org/10.1016/j.cub.2014.05.029

Dai X, Xu Y, Ma Q et al (2007) Overexpression of an r1r2r3 myb gene, osmyb3r-2, increases tolerance to freezing, drought, and salt stress in transgenic arabidopsis. Plant Physiol 143(4):1739-1751

Dong J, Cao L, Zhang X et al (2021) An r2r3-myb transcription factor rmmyb108 responds to chilling stress of rosa multiflora and conferred cold tolerance of arabidopsis. Front Plant Sci 12:696919. https://doi.org/10.3389/fpls.2021.696919

Dou TX, Hu CH, Sun XX et al (2016) Mpmybs3 as a crucial transcription factor of cold signaling confers the cold tolerance of banana. Plant Cell Tissue Organ Cult 125(1):93-106. https://doi.org/10.1007/s11240-015-0932-y

Fang L, Wang Z, Su L, Gong L, Xin H (2024) Vitis myb14 confer cold and drought tolerance by activating lipid transfer protein genes expression and reactive oxygen species scavenge. Gene 890:147792. https://doi.org/10.1016/j.gene.2023.147792

Gao Q, Yi L, Ge R et al (2025) A novel orphan gene csog3 drives csmyb44-dependent cold tolerance in tea plant. Tree Physiol. https://doi.org/10.1093/treephys/tpaf128

Ge W, Luo M, Sun H et al (2022) The camyb340 transcription factor induces chilling injury in post-harvest bell pepper by inhibiting fatty acid desaturation. The Plant journal : for cell and molecular biology 111(3):800-818. https://doi.org/10.1111/tpj.15854

Hong YQ, Ahmad N, Zhou Y et al (2023) The ctmyb63-ctu-box1-ctuch1 module regulates cold tolerance and hydroxysafflor yellow a accumulation in carthamus tinctorius. Ind Crops Prod 202. https://doi.org/10.1016/j.indcrop.2023.117088

Hou D, Cheng Z, Xie L et al (2018) The r2r3myb gene family in phyllostachys edulis: genome-wide analysis and identification of stress or development-related r2r3mybs. Front Plant Sci 9:738. https://doi.org/10.3389/fpls.2018.00738

Huang P, Ding Z, Duan M et al (2023) Oslux confers rice cold tolerance as a positive regulatory factor. International journal of molecular sciences 24(7). https://doi.org/10.3390/ijms24076727

Jiang H, Zhou L, Gao H, Wang X, Li Z, Li Y (2022) The transcription factor mdmyb2 influences cold tolerance and anthocyanin accumulation by activating sumo e3 ligase mdsiz1 in apple. Plant Physiol 189(4):2044-2060. https://doi.org/10.1093/plphys/kiac211

Jiao C, Wei Y, Dong J (2023) Ppmyb105 inhibits chilling injury by regulating ppmsra1 in peach fruit. Plant Cell Rep 42(10):1557-1569. https://doi.org/10.1007/s00299-023-03047-0

Jin R, Muhammad T, Jia C et al (2025) Overexpression of r2r3-myb type transcription factor spmyb1 enhances cold and drought tolerance in tomato. Plant physiology and biochemistry : PPB 229(Pt A):110326. https://doi.org/10.1016/j.plaphy.2025.110326

Kim SH, Kim HS, Bahk S et al (2017) Phosphorylation of the transcriptional repressor myb15 by mitogen-activated protein kinase 6 is required for freezing tolerance in arabidopsis. Nucleic Acids Res 45(11):6613-6627. https://doi.org/10.1093/nar/gkx417

Lee HG, Seo PJ (2015) The myb96-hhp module integrates cold and abscisic acid signaling to activate the cbf-cor pathway in arabidopsis. The Plant journal : for cell and molecular biology 82(6):962-977. https://doi.org/10.1111/tpj.12866

Li K, Lu Y, Li J et al (2025) Synergistic interaction between vymyb24 from chinese wild grape vitis yeshanensis and cytokinin enhances cold tolerance in transgenic tobacco. Plants (Basel, Switzerland) 14(24). https://doi.org/10.3390/plants14243777

Li M, Lin L, Zhang Y, Sui N (2019) Zmmyb31, a r2r3-myb transcription factor in maize, positively regulates the expression of cbf genes and enhances resistance to chilling and oxidative stress. Mol Biol Rep 46(4):3937-3944. https://doi.org/10.1007/s11033-019-04840-5

Li W, Li P, Chen H et al (2023) Overexpression of a fragaria vesca 1r-myb transcription factor gene (fvmyb114) increases salt and cold tolerance in arabidopsis thaliana. International journal of molecular sciences 24(6). https://doi.org/10.3390/ijms24065261

Li W, Wei Y, Zhang L et al (2023) Fvmyb44, a strawberry r2r3-myb transcription factor, improved salt and cold stress tolerance in transgenic arabidopsis. Agronomy

13(4):1051

Li W, Zhong J, Zhang L et al (2022) Overexpression of a fragaria vesca myb transcription factor gene (fvmyb82) increases salt and cold tolerance in arabidopsis thaliana. International journal of molecular sciences 23(18). https://doi.org/10.3390/ijms231810538

Li X, Jia J, Zhao P et al (2020) Lcmyb4, an unknown function transcription factor gene from sheepgrass, as a positive regulator of chilling and freezing tolerance in transgenic arabidopsis. BMC Plant Biol 20(1):238. https://doi.org/10.1186/s12870-020-02427-y

Li Y, Lin M, Zhang Q et al (2024) Overexpression of the kiwifruit transcription factor aamyb44 decreases the cold tolerance in arabidopsis thaliana. Plants (Basel, Switzerland) 13(22). https://doi.org/10.3390/plants13223126

Li Z, Zhou Y, Liang H et al (2023) Mamyb13 is involved in response to chilling stress via activating expression of vlcfas and phenylpropanoids biosynthesis-related genes in postharvest banana fruit. Food Chem 405(Pt B):134957. https://doi.org/10.1016/j.foodchem.2022.134957

Lian WH, Sun TX, Meng XY et al (2021) Overexpression of the panax ginseng myb4 gene enhances stress tolerance in transgenic arabidopsis thaliana. Biol Plant 65:27-38. https://doi.org/10.32615/bp.2020.164

Liao Y, Zou H, Wang H et al (2008) Soybean gmmyb76, gmmyb92, and gmmyb177 genes confer stress tolerance in transgenic arabidopsis plants. Cell Res 18(10):1047-1060. https://doi.org/10.1038/cr.2008.280

Liu W, Ou Q, Feng M et al (2025) Cold-induced promoter methylation attenuates scmyb7-mediated repression of the cbf pathway: a proposed mechanism for sugarcane cold adaptation. Plant, cell & environment 48(12):8973-8984. https://doi.org/10.1111/pce.70180

Liu W, Wang T, Wang Y, Liang X, Han J, Han D (2023) Mbmybc1, a m. Baccata myb transcription factor, contribute to cold and drought stress tolerance in transgenic arabidopsis. Front Plant Sci 14:1141446. https://doi.org/10.3389/fpls.2023.1141446

Liu Z, Qiao Z, Ren Y et al (2025) Myb20, an r2r3-type myb transcription factor, negatively regulates salt and cold stress tolerance in pears. Plant physiology and biochemistry : PPB 229(Pt A):110065. https://doi.org/10.1016/j.plaphy.2025.110065

Luo D, Raza A, Cheng Y, Zou X, Lv Y (2023) Cloning and functional characterization of cold-inducible myb-like 17 transcription factor in rapeseed (brassica napus l.). International journal of molecular sciences 24(11). https://doi.org/10.3390/ijms24119514

Luo Y, Wang Y, Li X et al (2024) Transcription factor dgmyb recruits h3k4me3 methylase to dgperoxidase to enhance chrysanthemum cold tolerance. Plant Physiol 194(2):1104-1119. https://doi.org/10.1093/plphys/kiad479

Lv Y, Yang M, Hu D et al (2017) The osmyb30 transcription factor suppresses cold tolerance by interacting with a jaz protein and suppressing β-amylase expression. Plant Physiol 173(2):1475-1491. https://doi.org/10.1104/pp.16.01725

Ma X, Yu Y, Jia J, Li Q, Gong Z (2022) The pepper myb transcription factor camyb306 accelerates fruit coloration and negatively regulates cold resistance. Sci Hortic 295:110892. https://doi.org/10.1016/j.scienta.2022.110892

Mahmoud LM, Killiny N, Dutt M (2024) Physiological and molecular responses of 'hamlin' sweet orange trees expressing the vvmyba1 gene under cold stress conditions. Planta 260(3):67. https://doi.org/10.1007/s00425-024-04496-x

Mao X, Jia D, Li A et al (2011) Transgenic expression of tamyb2a confers enhanced tolerance to multiple abiotic stresses in arabidopsis. Funct Integr Genomics 11(3):445-465. https://doi.org/10.1007/s10142-011-0218-3

Meng C, Sui N (2019) Overexpression of maize myb-if35 increases chilling tolerance in arabidopsis. Plant physiology and biochemistry : PPB 135:167-173. https://doi.org/10.1016/j.plaphy.2018.11.038

Meng X, Yin B, Feng HL, Zhang S, Liang XQ, Meng QW (2014) Overexpression of r2r3-myb gene leads to accumulation of anthocyanin and enhanced resistance to chilling and oxidative stress. Biol Plant 58(1):121-130. https://doi.org/10.1007/s10535-013-0376-3

Nie SM, Zhao RZ, Yang WW, Li JJ, Wang D (2024) Overexpression of slmyb1r1 improves chilling stress tolerance in tomato. Sci Hortic 338. https://doi.org/10.1016/j.scienta.2024.113662

Pasquali G, Biricolti S, Locatelli F, Baldoni E, Mattana M (2008) Osmyb4 expression improves adaptive responses to drought and cold stress in transgenic apples. Plant Cell Rep 27(10):1677-1686. https://doi.org/10.1007/s00299-008-0587-9

Peng X, Liu H, Wang D, Shen S (2016) Genome-wide identification of the jatropha curcas myb family and functional analysis of the abiotic stress responsive gene jcmyb2. BMC Genomics 17:251. https://doi.org/10.1186/s12864-016-2576-7

Qin T, Zhang M, Yi X et al (2026) Rsmyb90, a r2r3-myb transcription factor, plays a positive role in regulating low temperature stress in radish. Plant biology (Stuttgart, Germany) 28(1):69-78. https://doi.org/10.1111/plb.70118

Ruan M, Guo X, Wang B et al (2017) Genome-wide characterization and expression analysis enables identification of abiotic stress-responsive myb transcription factors in cassava (manihot esculenta). J Exp Bot 68(13):3657-3672. https://doi.org/10.1093/jxb/erx202

Shingote PR, Kawar PG, Pagariya MC, Muley AB, Babu KH (2020) Isolation and functional validation of stress tolerant eamyb18 gene and its comparative physio-biochemical analysis with transgenic tobacco plants overexpressing somyb18 and ssmyb18. 3 Biotech 10(5):225. https://doi.org/10.1007/s13205-020-02197-2

Shingote PR, Kawar PG, Pagariya MC, Rathod PR, Kharte SB (2017) Ectopic expression of ssmyb18, a novel myb transcription factor from saccharum spontaneum augments salt and cold tolerance in tobacco. Sugar Tech 19(3):270-282. https://doi.org/10.1007/s12355-016-0466-6

Song P, Yang R, Jiao K et al (2024) Fvmyb108, a myb gene from fragaria vesca, positively regulates cold and salt tolerance of arabidopsis. International journal of molecular sciences 25(6). https://doi.org/10.3390/ijms25063405

Su L, Li J, Liu D et al (2014) A novel myb transcription factor, gmmybj1, from soybean confers drought and cold tolerance in arabidopsis thaliana. Gene 538(1):46-55. https://doi.org/10.1016/j.gene.2014.01.024

Sun X, Matus JT, Wong DCJ et al (2018) The garp/myb-related grape transcription factor aquilo improves cold tolerance and promotes the accumulation of raffinose family oligosaccharides. J Exp Bot 69(7):1749-1764. https://doi.org/10.1093/jxb/ery020

Tian Y, Peng K, Ma X et al (2023) Overexpression of tamyb4 confers freezing tolerance in arabidopsis thaliana. International journal of molecular sciences 24(13). https://doi.org/10.3390/ijms241311090

Wang B, Wang G, Wang YK et al (2024) A cold-inducible myb like transcription factor, cshho2, positively regulates chilling tolerance of cucumber fruit by enhancing csgr-rbp3 expression. Postharvest Biol Technol 218. https://doi.org/10.1016/j.postharvbio.2024.113172

Wang CC, Liang C, Li HY et al (2024) Over-expression of sgrve1 from fine-stem stylo (stylosanthes guianensis var. Intermedia) up-regulates cbf gene expression and enhances cold tolerance in arabidopsis. Plant Cell Tissue Organ Cult 158(2). https://doi.org/10.1007/s11240-024-02838-x

Wang H, Shi S, Luo G et al (2024) Heterologous expression of a potential 'paulownia fortunei' myb factor gene, pfmyb90, improves salt and cold tolerance in arabidopsis. Plants (Basel, Switzerland) 14(1). https://doi.org/10.3390/plants14010024

Wang M, Hao J, Chen X, Zhang X (2020) Slmyb102 expression enhances low-temperature stress resistance in tomato plants. PeerJ 8:e10059. https://doi.org/10.7717/peerj.10059

Wang R, Cao Z, Hao Y (2014) Overexpression of a r2r3 myb gene mdsimyb1 increases tolerance to multiple stresses in transgenic tobacco and apples. Physiol Plant 150(1):76-87. https://doi.org/10.1111/ppl.12069

Wang Y, Mao Z, Jiang H, Zhang Z, Chen X (2019) A feedback loop involving mdmyb108l and mdhy5 controls apple cold tolerance. Biochem Biophys Res Commun 512(2):381-386. https://doi.org/10.1016/j.bbrc.2019.03.101

Wu RG, Wang Y, Wu T, Xu XF, Han ZH (2017) Mdmyb4, an r2r3-type myb transcription factor, plays a crucial role in cold and salt stress in apple calli. J Am Soc Hortic Sci 142(3):209. https://doi.org/10.21273/JASHS04030-17

Wu RG, Wang Y, Wu T, Xu XF, Han ZH (2018) Functional characterisation of mdmyb44 as a negative regulator in the response to cold and salt stress in apple calli. J Hortic Sci Biotechnol 93(4):347-355. https://doi.org/10.1080/14620316.2017.1373038

Xiao J, Wang D, Liang L et al (2024) Camyb80 enhances the cold tolerance of pepper by directly targeting capoa1. Hortic Res 11(10):uhae219. https://doi.org/10.1093/hr/uhae219

Xie Y, Chen P, Yan Y et al (2018) An atypical r2r3 myb transcription factor increases cold hardiness by cbf-dependent and cbf-independent pathways in apple. The New phytologist 218(1):201-218. https://doi.org/10.1111/nph.14952

Xin X, Wang S, Pan Y et al (2025) Myb transcription factor cdc5 activates cbf3 expression to positively regulate freezing tolerance via cooperating with ice1 and histone modification in arabidopsis. Plant, cell & environment 48(1):97-108. https://doi.org/10.1111/pce.15144

Xing C, Liu Y, Zhao L, Zhang S, Huang X (2019) A novel myb transcription factor regulates ascorbic acid synthesis and affects cold tolerance. Plant, cell & environment 42(3):832-845. https://doi.org/10.1111/pce.13387

Xu H, Yang G, Zhang J et al (2018) Overexpression of a repressor mdmyb15l negatively regulates anthocyanin and cold tolerance in red-fleshed callus. Biochem Biophys Res Commun 500(2):405-410. https://doi.org/10.1016/j.bbrc.2018.04.088

Yang A, Dai X, Zhang W (2012) A r2r3-type myb gene, osmyb2, is involved in salt, cold, and dehydration tolerance in rice. J Exp Bot 63(7):2541-2556. https://doi.org/10.1093/jxb/err431

Yang J, Yu S, Shi GF et al (2022) Comparative analysis of r2r3-myb transcription factors in the flower of iris laevigata identifies a novel gene regulating tobacco cold tolerance. Plant biology (Stuttgart, Germany) 24(6):1066-1075. https://doi.org/10.1111/plb.13452

Yang X, Luo Y, Bai H et al (2022) Dgmyb2 improves cold resistance in chrysanthemum by directly targeting dggpx1. Hortic Res 9. https://doi.org/10.1093/hr/uhab028

Yao C, Li W, Liang X et al (2022) Molecular cloning and characterization of mbmyb108, a malus baccata myb transcription factor gene, with functions in tolerance to cold and drought stress in transgenic arabidopsis thaliana. International journal of molecular sciences 23(9). https://doi.org/10.3390/ijms23094846

Yao C, Li X, Li Y et al (2022) Overexpression of a malus baccata myb transcription factor gene mbmyb4 increases cold and drought tolerance in arabidopsis thaliana. International journal of molecular sciences 23(3). https://doi.org/10.3390/ijms23031794

Yao L, Yang B, Xian B et al (2020) The r2r3-myb transcription factor bnamyb111l from rapeseed modulates reactive oxygen species accumulation and hypersensitive-like cell death. Plant physiology and biochemistry : PPB 147:280-288. https://doi.org/10.1016/j.plaphy.2019.12.027

Yong Y, Zhang Y, Lyu Y (2019) A myb-related transcription factor from lilium lancifolium l. (Llmyb3) is involved in anthocyanin biosynthesis pathway and enhances multiple abiotic stress tolerance in arabidopsis thaliana. International journal of molecular sciences 20(13). https://doi.org/10.3390/ijms20133195

Yu J, Lei B, Zhao H et al (2022) Cloning, characterization and functional analysis of ntmyb306a gene reveals its role in wax alkane biosynthesis of tobacco trichomes and stress tolerance. Front Plant Sci 13:1005811. https://doi.org/10.3389/fpls.2022.1005811

Yu Q, Zheng Q, Liu C et al (2025) Phosphorylation-dependent vamyb4a regulates cold stress in grapevine by inhibiting vapif3 and activating vacbf4. Plant Physiol 197(2). https://doi.org/10.1093/plphys/kiaf035

Yu S, Li P, Liu H et al (2025) A cca1-like myb subfamily member csmyb128 participates in chilling sensitivity and cold tolerance in tea plants (camellia sinensis). Int J Biol Macromol 294:139473. https://doi.org/10.1016/j.ijbiomac.2025.139473

Yue R, Li Y, Qi Y et al (2025) Divergent myb paralogs determine spatial distribution of linalool mediated by ja and dna demethylation participating in aroma formation and cold tolerance of tea plants. Plant Biotechnol J 23(5):1455-1475. https://doi.org/10.1111/pbi.14598

Zhai H, Bai X, Zhu Y et al (2010) A single-repeat r3-myb transcription factor mybc1 negatively regulates freezing tolerance in arabidopsis. Biochem Biophys Res Commun 394(4):1018-1023. https://doi.org/10.1016/j.bbrc.2010.03.114

Zhang C, Liu H, Zhang X et al (2020) Vcmyb4a, an r2r3-myb transcription factor from vaccinium corymbosum, negatively regulates salt, drought, and temperature stress. Gene 757:144935. https://doi.org/10.1016/j.gene.2020.144935

Zhang H, Hu Y, Gu B, Cui X, Zhang J (2022) Vamyb44 transcription factor from chinese wild vitis amurensis negatively regulates cold tolerance in transgenic arabidopsis thaliana and v. Vinifera. Plant Cell Rep 41(8):1673-1691. https://doi.org/10.1007/s00299-022-02883-w

Zhang J, Yin X, Li H et al (2020) Ethylene response factor39-myb8 complex regulates low-temperature-induced lignification of loquat fruit. J Exp Bot 71(10):3172-3184. https://doi.org/10.1093/jxb/eraa085

Zhang L, Jiang X, Liu Q et al (2020) The hy5 and myb15 transcription factors positively regulate cold tolerance in tomato via the cbf pathway. Plant, cell & environment 43(11):2712-2726. https://doi.org/10.1111/pce.13868

Zhang L, Liu G, Zhao G et al (2014) Characterization of a wheat r2r3-myb transcription factor gene, tamyb19, involved in enhanced abiotic stresses in arabidopsis. Plant & cell physiology 55(10):1802-1812. https://doi.org/10.1093/pcp/pcu109

Zhang L, Zhao G, Xia C, Jia J, Liu X, Kong X (2012) Overexpression of a wheat myb transcription factor gene, tamyb56-b, enhances tolerances to freezing and salt stresses in transgenic arabidopsis. Gene 505(1):100-107. https://doi.org/10.1016/j.gene.2012.05.033

Zhang N, Zhang H, Bai B et al (2025) Jasmonic acid activates transcription factor slmyb13 to enhance cold resistance in tomato. Plant, cell & environment 48(10):7332-7347. https://doi.org/10.1111/pce.70030

Zhang QY, Yu JQ, Wang JH, Hu DG, Hao YJ (2017) Functional characterization of mdmyb73 reveals its involvement in cold stress response in apple calli and arabidopsis. J Integr Agric 16(10):2215-2221. https://doi.org/10.1016/S2095-3119(17)61723-4

Zhang WH, Zhang NY, Qin Q et al (2024) Rmmyb44 confers resistance to chilling, drought, and salt stress in both rosa multiflora and tobacco. Agriculture (Basel) 14(8). https://doi.org/10.3390/agriculture14081212

Zhang Y, Xu Y, Xing W et al (2023) Identification of the passion fruit (passiflora edulis sims) myb family in fruit development and abiotic stress, and functional analysis of pemyb87 in abiotic stresses. Front Plant Sci 14:1124351. https://doi.org/10.3389/fpls.2023.1124351

Zhang Z, Hu X, Zhang Y et al (2016) Opposing control by transcription factors myb61 and myb3 increases freezing tolerance by relieving c-repeat binding factor suppression. Plant Physiol 172(2):1306-1323. https://doi.org/https://doi.org/10.1104/pp.16.00051

Zhao YQ, Liu Z, Zhao LY et al (2026) Transcription factor ppmyb73 inhibits ppppo1 transcription and flesh browning in hydrogen sulfide-mitigated chilling injury of peach fruit. Postharvest Biol Technol 232. https://doi.org/10.1016/j.postharvbio.2025.114004

Zhou L, Li R, Yang X et al (2025) Interaction of r2r3-myb transcription factor egmyb111 with aba receptors enhances cold tolerance in oil palm. Int J Biol Macromol 305(Pt 2):141223. https://doi.org/10.1016/j.ijbiomac.2025.141223

Zhou L, Yarra R, Yang Y, Liu Y, Yang M, Cao H (2022) The oil palm r2r3-myb subfamily genes egmyb111 and egmyb157 improve multiple abiotic stress tolerance in transgenic arabidopsis plants. Plant Cell Rep 41(2):377-393. https://doi.org/10.1007/s00299-021-02814-1

Zhou ML, Wang CL, Qi LP et al (2015) Ectopic expression of fagopyrum tataricum ftmyb12 improves cold tolerance in arabidopsis thaliana. J Plant Growth Regul 34(2):362-371. https://doi.org/10.1007/s00344-014-9472-7

Zhou X, Lei D, Yao W et al (2024) A novel r2r3-myb transcription factor pbmyb1l of pyrus bretschneideri regulates cold tolerance and anthocyanin accumulation. Plant Cell Rep 43(2):34. https://doi.org/10.1007/s00299-023-03117-3

Zhou Z, Wei X, Lan H (2023) Cgmyb1, an r2r3-myb transcription factor, can alleviate abiotic stress in an annual halophyte chenopodium glaucum. Plant physiology and biochemistry : PPB 196:484-496. https://doi.org/10.1016/j.plaphy.2023.01.055

Zhu C, Yang X, Chen W et al (2024) Wd40 protein osttg1 promotes anthocyanin accumulation and cbf transcription factor-dependent pathways for rice cold tolerance. Plant Physiol 197(1). https://doi.org/10.1093/plphys/kiae604

Zhu L, Li SS, Ma QY et al (2023) An acer palmatum r2r3-myb gene, apmyb77, confers freezing and drought tolerance in arabidopsis thaliana. J Plant Growth Regul 42(2):1017-1030. https://doi.org/10.1007/s00344-022-10611-7
